# Primary care practice-based care management for chronically ill patients (PraCMan): study protocol for a cluster randomized controlled trial [ISRCTN56104508]

**DOI:** 10.1186/1745-6215-12-163

**Published:** 2011-06-29

**Authors:** Tobias Freund, Frank Peters-Klimm, Justine Rochon, Cornelia Mahler, Jochen Gensichen, Antje Erler, Martin Beyer, Annika Baldauf, Ferdinand M Gerlach, Joachim Szecsenyi

**Affiliations:** 1 Department of General Practice and Health Services Research, University Hospital Heidelberg, Voßstrasse 2, 69115 Heidelberg, Germany; 2 Institute of Medical Biometry and Informatics, University of Heidelberg, Im Neuenheimer Feld 305, 69120 Heidelberg, Germany; 3 Institute of General Practice, Friedrich Schiller University Jena, Bachstraße 18, 07743 Jena, Germany; 4 Institute of General Practice, Johann Wolfgang Goethe University Frankfurt, Theodor-Stern-Kai 7, 60590 Frankfurt am Main, Germany

## Abstract

**Background:**

Care management programmes are an effective approach to care for high risk patients with complex care needs resulting from multiple co-occurring medical and non-medical conditions. These patients are likely to be hospitalized for a potentially "avoidable" cause. Nurse-led care management programmes for high risk elderly patients showed promising results. Care management programmes based on health care assistants (HCAs) targeting adult patients with a high risk of hospitalisation may be an innovative approach to deliver cost-efficient intensified care to patients most in need.

**Methods/Design:**

PraCMan is a cluster randomized controlled trial with primary care practices as unit of randomisation. The study evaluates a complex primary care practice-based care management of patients at high risk for future hospitalizations. Eligible patients either suffer from type 2 diabetes mellitus, chronic obstructive pulmonary disease, chronic heart failure or any combination. Patients with a high likelihood of hospitalization within the following 12 months (based on insurance data) will be included in the trial.

During 12 months of intervention patients of the care management group receive comprehensive assessment of medical and non-medical needs and resources as well as regular structured monitoring of symptoms. Assessment and monitoring will be performed by trained HCAs from the participating practices. Additionally, patients will receive written information, symptom diaries, action plans and a medication plan to improve self-management capabilities. This intervention is addition to usual care.

Patients from the control group receive usual care.

Primary outcome is the number of all-cause hospitalizations at 12 months follow-up, assessed by insurance claims data. Secondary outcomes are health-related quality of life (SF12, EQ5D), quality of chronic illness care (PACIC), health care utilisation and costs, medication adherence (MARS), depression status and severity (PHQ-9), self-management capabilities and clinical parameters. Data collection will be performed at baseline, 12 and 24 months (12 months post-intervention).

**Discussion:**

Practice-based care management for high risk individuals involving trained HCAs appears to be a promising approach to face the needs of an aging population with increasing care demands.

**Trial registration:**

Current Controlled Trials ISRCTN56104508

## Background

Healthcare systems are challenged by an increasing number of patients with multiple chronic conditions [1]. Individuals with multiple chronic conditions are more likely to be at risk for functional impairment [2] and adverse drug events [3]. Self management capabilities decline with an increasing number of co-occurring medical conditions [4]. In addition, medical care for patients with multiple chronic conditions is often fragmented by poor coordination between different healthcare providers [5]. These patients are more likely to be hospitalised for a potentially 'avoidable' cause (e.g., unmanaged exacerbation, intermittent infection or falls, imperfect transitional care), leading to suboptimal health outcomes [6] and substantial healthcare costs [7].

Particularly primary care faces a challenge to care for patients with multiple chronic conditions on the background of limited human and restricted financial resources [8,9]. Different suggestions are available to take up the challenge by re-organising the delivery of chronic illness care [10,11]. Based on these concepts, care management interventions have been developed and evaluated focusing on patients with multiple chronic conditions. These interventions share four core elements [12]: (a) comprehensive assessment of patients' medical and non-medical needs and resources, (b) implementation and monitoring of individualised, evidence-based care plans, (c) coordination of services between providers of medical and social care, and (d) enhancement of self-management capabilities among patients and caregivers. Despite positive effects on quality of care and patients' quality of life, the effect on healthcare utilisation and costs remains heterogeneous [9]. Care management interventions have shown to be effective and efficient if they focus on patients with high risk of healthcare utilization [9,13].

It is crucial to identify the patients most likely to benefit from care management programs (case finding). Based on insurance claims data, so called predictive models can be used to identify patients at high risk for future health care utilization [14]. However, not all high risk patients are able and willing to participate in care management programs. Thus, case finding by predictive modelling should be complemented by the treating physician who can identify high risk patients most likely to participate in and benefit from care management [15].

In Germany, chronic heart failure (CHF), chronic obstructive pulmonary disease (COPD), and type 2 diabetes mellitus (DM) were among the 20 most frequent causes for hospital admission in 2009 [16]. All three conditions are 'ambulatory care sensitive conditions' (ACSC), meaning that primary care plays an important role in preventing hospital admissions for these conditions [17]. These hospitalisations may be avoidable by coordinated and structured chronic care. Based on exploratory studies, we developed a primary care practice-based care management intervention for patients suffering from any of these index conditions with a high likelihood of hospitalization as predicted by a statistical model [18]. Nearly all of these patients suffer from significant co-morbidities [19,20]. The care management intervention is designed to account for the complex care needs resulting from these co-morbidities.

Chronic care in Germany is mainly delivered by small primary care practices: The practice team usually consists of one or two physicians (general practitioner or general internist) and a small number of healthcare assistants (HCAs), who perform few clinical tasks. HCAs are trained in a three-year part-time curriculum in practice (3920 hours) and vocational school (840 hours). Despite some recent attempts to involve HCAs in chronic care [21], their work is mostly focused on clerical work (including reception) and routine tasks like blood sampling or electrocardiogram recording. However, recent trials on primary care-based disease-specific case management interventions involving trained HCAs showed promising results in patients with osteoarthritis, [22], depression [23] and systolic heart failure [24]. Moreover, practice teams experienced the expanded role of HCAs as valuable resource to enhance the provision of chronic care [25-27].

### Novel aspects of PraCMan

Whereas international research on care management has mainly focused on nurse-led programmes, evidence on the potential role and effects of HCAs in chronic care is scarce. As cost-efficiency of intensified care programs appears to be essential for further implementation, HCA-based programmes may offer an opportunity to deliver intensified care with lower staff costs.

The limited medical education of HCAs could be a potential problem in the care for high-risk patients with multiple chronic conditions and needs to be addressed when designing HCA-based programs. Therefore, PraCMan focuses on three index conditions (DM, CHF, COPD) and distinct comorbidity patterns which we could explore to be common in a population of patients at high risk for future hospitalization [28]. Furthermore, structured written protocols for assessment and monitoring are feasible tools for HCA-based care management programs [29,30]. We designed a modular concept of structured monitoring lists that can be applied either by phone or in the practice. This concept accounts for index conditions as well as for frequent comorbidities.

As case finding has shown to be crucial for the success of intensified care programs, we selected a statistical model based on insurance claims data that predicts future hospitalization instead of costs. A significant proportion of these hospitalizations account for "actionable costs" as they appear to be potentially avoidable. This predictive model proved to be useful to identify patients in need for intensified care [15].

Primary care practice-based care management for patients with either DM, COPD or CHF and a high predicted risk for future hospitalization involving HCAs is a promising approach for effective and cost-efficient intensified care for the patients most in need. This paper describes aim and methods of the PraCMan trial.

## Methods/Design

### Primary objective

The primary objective of this study is to determine whether a primary care practice-based care management intervention will reduce the number of all-cause hospitalizations as compared to standard care. The intervention focuses on patients at high risk for future hospitalizations suffering from DM, COPD, CHF or any combination of these index conditions. The intervention is additional to usual care.

### Secondary objectives

We will explore effects on re-admissions, disease-specific admissions, hospital days, mortality, health-related quality of life, self-management capabilities, medication adherence, physical outcome parameters (e.g. NYHA, number of exacerbations, depressive symptoms), patients' satisfaction with medical care, health service utilization and costs. Besides, we will explore the effects of the intervention on work satisfaction of primary care physicians (PCPs) and HCAs as well as practice organization.

### Study design

**Figure 1:**
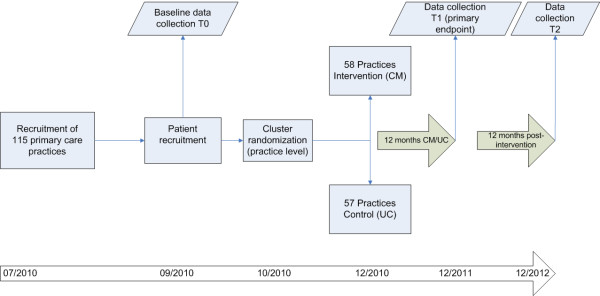
**Design of the PraCMan trial**. This figure shows design and timeframe of the PraCMan trial.

The study is designed as a prospective two-armed open cluster randomized controlled trial. Randomization at the level of primary care practice was chosen to prevent contamination between intervention and control patients. Blinding of either patients or practice teams was not possible due to the character of the intervention. However, observers will be blinded during data collection regarding primary and secondary endpoints. Patients were randomized in clusters of 15-20 patients per practice (see Figure 1). Each patient was assigned to a care management team (CM team) consisting of one PCP and one HCA. All primary and secondary endpoints will be measured at either practice or patient level at baseline (T0), at 12 months (T1) and 24 months (T2) after randomization.

### Sample size calculation

Sample size was calculated by using the primary endpoint, i.e. the number of all-cause hospitalizations per patient in the 12 months intervention period. Based on data from our pilot study, we estimated a mean number of 0.7 all-cause hospitalizations per patient per year with a standard deviation of 1 [18]. Based on these estimates, a total of 1602 patients (801 per arm) would be required to detect a between-group difference of 0.14 (20% absolute reduction) with a power of 80% and by using a two-sample *t*-test at a 5% significance level (two-sided). An intracluster correlation (ICC) of 0.01 at practice level could be estimated on the basis of data from a comparable intervention study [24]. Assuming an ICC of 0.01 and an average cluster size of 17, we estimated a design effect of D = 1 + (17 - 1) × 0.01 = 1.16. Taking this design effect into account, a total of 110 practices and 1870 patients (55 practices and 935 patients per arm) will be required. Recent intervention studies reported drop-out rates of 6% [31] and 10% [24], respectively. Therefore, assuming a drop out rate of 15%, we aimed to include 2,210 patients and 130 practices.

### Recruitment of practice sites and patients

PCPs were eligible for the study if they were participating in the primary care-centred care contract of the General Regional Health Fund (AOK). Participation in the primary care (PC)-centred care contract is voluntary for physicians and patients. Care within this contract implies a gate keeping role of the PCP, PCP training and feedback regarding evidence based pharmacotherapy and patient benefits such as reduced waiting times in the practice and late afternoon consultation hours.

We invited eligible PCPs in the Federal State of Baden-Württemberg (Germany) by a formal letter to participate in the study. 132 PCPs from 115 practices gave their written consent and were randomized into the study groups. 234 PCPs gave information before randomization that they decline to participate due to lack of time (n = 129), practice organization (n = 16), unattractive reimbursement (n = 7), no interest (n = 2), concerns about funding source (n = 1), health status of PCP (n = 4) and plans about giving-up practice in near future (n = 7). 63 PCPs did not further specify their reason for declined participation, whereas 5 PCPs were not able to recruit study patients and withdrew participation before randomization.

From the study coordinating centre PCPs received a list of potentially eligible patients based on inclusion and exclusion criteria (see below). After validation of their eligibility patients were recruited by participating PCPs who were blinded to their allocation to the study groups. Patients not willing to participate were asked to give a reason for their decision (on a voluntary basis). PCPs were asked to document patients screened, asked and not included as well as the reason for inclusion and exclusion.

### Randomization

Practices were randomly allocated to care management or usual care in the ratio of 1:1. We performed block randomisation with variable block lengths to ensure study groups of approximately equal size. As population density has shown to have significant impact on hospitalizations [32], cluster randomization was stratified according to population density of the regions of participating practice sites based on a map provided by the Federal Agency of Regional Development Planning [33]. We used computer generated randomization lists (SAS Version 9.2). Separate randomisation lists were prepared for urban and rural practices. Central randomisation was performed by a research assistant who was not involved in the project. PCPs allocation to either intervention or control group remained concealed until patient recruitment in the practice was fully completed. After this, PCPs were informed about their allocation with an official letter from the study coordinating centre.

### Inclusion/Exclusion criteria

#### Practice inclusion and exclusion criteria

Eligible primary care practices had to fulfil the following criteria: Participation in the PC-centred care contract of the AOK-Baden-Württemberg, at least one PCP (e.g. general practitioner or general internist) who was willing to participate in the study, at least one participating HCA, and the ability to perform on-site spirometry and home visits.

Practices which quit participation in the PC-centred care contract of the AOK-Baden-Württemberg during the study will be excluded. Participation in similar clinical trials (e.g. telemonitoring studies) is exempted for participating practices.

#### Patient inclusion and exclusion criteria

To be eligible for the study, patients had to suffer from at least one of the following index conditions: type 2 diabetes mellitus under medical treatment and/or chronic obstructive pulmonary disease under medical treatment and/or chronic heart failure with confirmed diagnosis by a cardiologist. Further inclusion criteria were: High risk for future hospitalization (i.e. predicted likelihood of hospitalization within the upper quartile of the total patient population) and age ≥ 18 years.

The likelihood of at least one hospitalization within the next 12 months (LOH) was calculated on the basis of pseudonymised insurance claims data of all AOK beneficiaries from the participating practices. The software package Case Smart Suite Germany 0.7 (Verisk Health, Munich, Germany) was used to compute LOH. The software calculates LOH on the basis of all ICD10-GM (German modification) inpatient and outpatient diagnosis codes, prior costs and hospital admissions as well as demographic data. All input variables were obtained for a pre-prediction interval of 18 months [15].

Patients with the following criteria were excluded: Active cancer disease (defined as cancer diagnosis under current treatment with radiotherapy or chemotherapy), moderate to severe dementia, permanent residency in a nursing home, participation in a concurrent clinical trial (involving telemonitoring studies), severe physical and mental disorders or other problems that hinder active participation in the intervention (e.g. Non-German speaking patients).

### Data collection

After obtaining written informed consent, patients were registered in the study coordinating centre of the Department of General Practice and Health Services Research Heidelberg (Germany) which is responsible for administration, coordination, data management and monitoring (includes database-set up and validation, data entry, coding, and query management).

Patients will be asked to fill in a pseudonymised paper-based questionnaire. PCPs will document additional data from patients' chart and assess the clinical status of the patient (e.g. dyspnoea, blood pressure). Patient questionnaire and chart review will be performed at baseline, 12-, and 24- month follow-up. In addition to these data sources, pseudonymised insurance claims data of participating patients will be obtained from the AOK including data on health service utilisation, medication, diagnosis and costs. All data from patient questionnaires, case report forms and insurance claims will be compiled.

### Outcome measures

#### Primary outcome

The number of all-cause hospitalizations per patient during 12 months of follow up will be determined on the basis of insurance claims data.

#### Secondary outcomes

Additional information regarding hospital admissions will be obtained from a chart review (planned/un-planned admission) and case report forms. Health-related quality of life will be assessed using the Short Form 12 Health Questionnaire (SF-12) and the EuroQol instrument EQ-5D (see table 1). Other secondary outcome parameters are: Patient Assessment of Chronic Illness Care (PACIC), Medication Adherence (MARS), depression (PHQ-9), self-management capabilities (European Heart Failure Selfcare Behaviour Scale, self-developed scales for DM- and COPD-related self management), physical activity (RAPA), activities of daily living (ADL/IADL), mortality, healthcare utilization (emergency department visits, practice visits, skilled nursing home days) and total healthcare costs. Additionally, clinical parameters will be assessed for all patients (blood pressure), DM-patients (HbA1c, number of (severe) hypoglycaemias, fasting glucose), COPD-patients (MRC dyspnoea sale, forced expiratory volume, number of exacerbations) and CHF-patients (NYHA, number of exacerbations). Qualitative (focus groups with PCPs, HCAs and patients) as well as quantitative (CM documents) process evaluation is planned in order to investigate how the PraCMan intervention is implemented into practice.

**Table 1:** Outcome parameter and instruments of the PraCMan trial

Outcome parameter	Instrument	Data source
**Primary outcome**		

All-cause hospitalizations	Data on admissions	IC

**Secondary outcomes**		

Sociodemographic data	Single items from German standard questionnaire [44]	PQ

Mortality	Patient chart	CRF/IC

Quality of Life	EuroQol (EQ-5D) [45]	PQ

	Short Form 12 Health Questionnaire (SF 12) [46]	PQ

Quality of Care	Patient Assessment of Chronic Illness Care (PACIC) [47]	PQ

Depression	Patient Health Questionnaire (PHQ9) [48]	PQ

Adherence	Medication Adherence Reporting Scale (MARS) [49]	PQ

Physical Activity	The Rapid Assessment of Physical Activity (RAPA) [50]	PQ

Smoking status	Self developed items	CRF

Self-management CHF	European Selfcare Behaviour Scale (EHFScB) [51]	PQ

Self-management COPD/DM	Self developed instrument	PQ

Medication regimen	Pharmacy data	IC

Healthcare costs	Data from hospital care, ambulatory care, nursing facilities, pharmacies, rehabilitation	IC

Activities of daily living	ADL [52] IADL [53]	CRF

Comorbidity	Cumulative Illness Rating Scale (CIRS) [54]	CRF

Home visits/Practice visits	Self developed items	CRF

CHF decompensation (CHF patients)	Self developed items	CRF

COPD exacerbation (COPD patients)	Self developed items	CRF

Hypoglycaemia (DM patients)	Self developed items	CRF

Body mass index	Weight, Height	CRF

Blood pressure (all patients)	Standardized Measurement in the practice	CRF

Fasting glucose	Patient chart	CRF

Hemoglobin-A1c	Patient chart	CRF

Dyspnoea (CHF and COPD patients)	Current NYHA-classification (CHF patients) or current MRC Dyspnoe Score (COPD patients) [55]	CRF

Forced expiratory volume (COPD patients)	Standardized Measurement in the practice	CRF

PQ: Patient questionnaire, CRF: Case Report Form, IC: Insurance claims data.

### Statistical analysis

Data will be analysed in accordance with the CONSORT statement and its extension for cluster randomised trials [34]. The primary analysis will be performed adhering to the intention-to-treat principle. An additional sensitivity analysis will be conducted on a per-protocol analysis set. Descriptive statistics will be used to summarise characteristics of both practices and patients. A multilevel modelling approach [35] will be applied to evaluate differences between the intervention group and the control group on the primary and all secondary outcomes. This approach will be used to account for the hierarchical structure of the data (i.e. patients nested within practices). Evaluations will include analyses of subgroups based on morbidity, health care utilization and predicted risk of future hospitalization. The effect of the intervention on the primary outcome will be tested at the two-sided significance level of 5%. The result will be presented as the difference between group means with the corresponding 95% confidence interval after adjustment for baseline characteristics. Only the result of this primary efficacy analysis will be interpreted in a confirmatory manner. Interim analyses are not planned. Statistical analyses will be carried out with SAS Version 9.2 (SAS Institute, Cary, NC, USA).

## Intervention

PraCMan is a complex care management intervention [36]. Based on the results of a series of exploratory studies [18] a multifaceted intervention was developed to reduce (avoidable) hospitalizations of high risk patients. The intervention consists of three elements:

### Assessment

At the beginning, all patients receive an assessment of medical and non-medical needs and resources using a structured protocol by a practice-based HCA. Contents are: Vaccination status (Influenza, *Streptococcus pneumonia*), allergies, nutritional problems, depression, falls, medication adherence, medication ("brown bag review"), pain, physical activity, smoking status, hearing/seeing problems, constipation, and financial/social situation. In accordance with patient-centred care patients will be asked to prioritise the three most important problems - no matter whether medical or non-medical [37]. The assessment can be performed at a home visit if patients are immobile. Patients with a high risk of falls (determined by the assessment) will receive a preventive home visit by their HCAs in order to identify and remove tripping hazards.

### Planning

At a first step, PCPs and HCAs will discuss the results of the assessment in order to identify needs that can be targeted by care management. These targets will be refined in collaboration with patients and caregivers, if applicable: Patients will be motivated to set long-term goals for CM. Patients and CM teams will then jointly define concrete steps (short-term goals) needed to achieve the long term goals. Self-efficacy to achieve these short term goals will be assessed using a 10-step Likert Scale ("How confident are you that you will take this step during the next XY weeks?"). A 0 displays "absolute no confidence", 10 displays "absolute confidence". If patients rate their self-efficacy lower than 7, concrete steps will be further discussed in order to set a more realistic short term goal. As part of care planning, all patients will receive a patient folder containing disease specific information leaflets, symptom diaries, action plans for self-managing acute exacerbations, a list of diagnosis and allergies, a current medication list, medical reports, lab results, and contact data of the PCP. Additionally, long and short term goals will be documented in the patient folder. The patient is encouraged to take the folder to each medical encounter. The first page of the folder contains a red box for hospital doctors prompting them to call the PCP at least 24 hours prior to discharge. Patients will be trained in the use of the patient folder during their first encounter with their HCA.

### Monitoring

Practice-based HCAs will deliver a regular telephone monitoring using the PraCMan monitoring list. Content and frequency of the monitoring will be determined by the PCP. A core module of monitoring items is fixed for all patients. Modules on DM, COPD, CHF, and depression could be selected for individual conditions. Frequency of monitoring is stratified by patients' risk for clinical deterioration: level 1 indicating 6-weekly contacts (low risk of acute deterioration of symptoms), level 2 indicating 3-weekly contacts (moderate risk of acute deterioration of symptoms) and level 3 triggering weekly contacts (high risk acute deterioration of symptoms, e.g. first days after discharge from an unplanned hospitalization).

The PraCMan monitoring list has been developed based on experiences in prior studies of our group and will be published elsewhere [29,30]. Item responses are colour-coded green, yellow, or red according to the urgency of the symptoms and signs presented by the patient which will guide the HCA-PCP interaction. Red-coloured answers require immediate contact to the PCP, orange-coloured answers require the patient to be seen within 24 hours and yellow-coloured responses are reported to the PCP within 3 days.

### Training of Case Management teams

Prior to the beginning of the intervention, all CM teams will be trained according to a team-based training curriculum. The curriculum was developed based on literature review, experiences from prior studies [23,24] and exploratory studies [18] and will be published elsewhere. Completion of a 36 hour training course (20 hours self-study, 16 hours interactive workshop) is mandatory for participating HCAs. PCPs will be invited to take part in an 8 hour workshop. PCPs and HCAs will be trained jointly in communication techniques and goal-setting in order to enhance communication within the CM-team.

### Control

Practice teams in the control group will continue to provide standard care in the context of the PC-centred care contract [38]. This involves gate keeping for enrolled patients as well as regular training in evidence-based guidelines through structured pharmacotherapy feedback in peer review groups [39]. As population-based disease management programs (DMPs) for DM, COPD and CHF (based on coronary heart disease) are part of routine care in Germany, patients may voluntarily participate in these disease specific care programs. German DMPs consist of regular follow up visits up to every three months. They include clinical examination, laboratory tests (e.g. HbA1c tests) and patient education [40]. However, essential elements of care management interventions like individualized assessment, care planning and frequent (symptom) monitoring are not routinely part of DMPs [41].

### Ethics and legal aspects

The study is being conducted in accordance with medical professional codex and the Helsinki Declaration as well as the ICH Guideline for Good Clinical Practice (GCP). The study is also in accordance with German Federal Data Security Law (BDSG). All professionals participating in the study oblige to adhere to the abovementioned declarations and laws. The study protocol was approved by the ethics committee of the University Hospital Heidelberg (S-232/2010) and by the ethics committee of the Federal Medical Association Baden-Wuerttemberg (B-F-2010-043) prior to the start of the study. The study is registered at http://www.controlled-trials.com (ISRCTN56104508).

### Timeframe of the study

Practice recruitment was performed between November 2009 and August 2010 (see Figure 1). Recruitment of eligible patients and baseline data collection started in September 2010. After cluster randomization, all CM-teams in intervention practices were trained prior to the beginning of the intervention in November and December 2010. Data collection for intermediate and final assessment of outcome parameters is scheduled for December 2011 (end of intervention) and December 2012 (1 year post intervention).

## Discussion

Primary care practice-based care management for chronically ill patients at high risk for hospitalisation involving trained HCAs may improve patients' needs and outcomes. HCAs have increasingly been recognised as an underexploited resource in chronic care [42]. In Germany, additional qualification programmes for HCAs have recently been installed [43]. The PraCMan training curriculum for HCAs is designed to complement these programs and could therefore be implemented into routine care. Provided that our intervention proves to be effective, this care program could be disseminated throughout PC-centred care contracts nationwide. We designed PraCMan to reduce health care costs by reducing the number of avoidable hospitalizations. Either budget neutrality or net savings would provide a rationale for health funds to offer this care management program, especially if it also improves quality of care, quality of life, and self management capabilities of patients.

## List of abbreviations

ACSC: Ambulatory care sensitive conditions; BDSG: Federal Data Security Law (= Bundesdatenschutzgesetz); CM: Care Management; CHF: chronic heart failure; COPD: Chronic obstructive pulmonary disease; DM: Type 2 diabetes mellitus; DMP: Disease management program; GCP: Good Clinical Practice; HCA: Health Care Assistant; LOH: Likelihood of hospitalization; PCP: Primary Care Physician.

## Competing interests

The authors declare that they have no competing interests.

## Authors' contributions

TF, FPK, CM, JG, AE, MB, AB and JS developed intervention and study protocol. JR contributed to the development of the study protocol. TF wrote the first draft of the manuscript. FPK, CM, JR, JG, AE, FMG and MB critically revised it. All authors read and approved the final manuscript.
